# Supplementary material for: Triple-Negative Breast Cancer with High Levels of Annexin A1 Expression Is Associated with Mast Cell Infiltration, Inflammation, and Angiogenesis
Source: Int J Mol Sci. 2019 Aug 27;20(17):4197. doi: 10.3390/ijms20174197 (PMC6747082; doi:10.3390/ijms20174197)
Supplement: Supplementary file 1 [file ijms-20-04197-s001.pdf]

Supplementary Table S1. Clinical factors and ANXA1 protein expression in 48 breast cancer cases.

Characteristics		Total (n=48)	ANXA1		P-value
			positive (n=17)	negative (n=31)	
Age					0.524
	<50	16	7	9	
	≧50	32	10	22	
pTMN stage					0.311
	I	18	5	13	
	IIA	13	3	10	
	IIB	16	8	8	
	III	1	1	0	
Lymph node metastasis					0.551
	positive	22	9	13	
	negative	26	8	18	
Histology					0.396
	Invasive ductal	41	16	25	
	Others	7	1	6	
Histological Grade					0.211
	1,2	23	6	17	
	3	21	10	11	
	not reported	4	1	3	
Lymphatic invasion					1
	positive	24	9	15	
	negative	21	7	14	
	not reported	3	1	2	
Venous invasion					0.131
	positive	10	6	4	
	negative	35	10	25	
	not reported	3	1	2	

Table1. Significant gene sets that associated with high expression of AR using Gene Set Enrichment Analysis (GSEA)(TCGA)

NAME	SIZE	ES	NES	NOM p-val	FDR q-val	FWER p-val
HALLMARK_UV_RESPONSE_DN	140	0.5521	2.0113	0.0000	0.0126	0.0210

1 ES, enrichment score; 2 NES, normalized enrichment score; 3 NOM-p-val, normalized p-value; 4 FDR q-val, false discovery rate q-value; 5 FWER p-val, family-wise error rate p-value

Table2. Significant gene sets that associated with low expression of AR using Gene Set Enrichment Analysis (GSEA)(TCGA)

NAME	SIZE	ES	NES	NOM p-val	FDR q-val	FWER p-val
HALLMARK_DNA_REPAIR	142	-0.5265	-2.0298	0.0000	0.0281	0.0150

1 ES, enrichment score; 2 NES, normalized enrichment score; 3 NOM-p-val, normalized p-value; 4 FDR q-val, false discovery rate q-value; 5 FWER p-val, family-wise error rate p-value

Supplementary Table S2. Clinical factors and ANXA1 mRNA expression in TCGA and METABRIC TNBC cohorts.

Characteristics		TCGA					METABRIC			
		Total	ANXA1		P-value		Total	ANXA1		P-value
		(n=173)	high (n=87)	low (n=86)			(n=313)	high (n=157)	low (n=156)	
Age					0.588					0.437
	<50	55	26	29			115	61	54	
	≧50	118	61	57			198	96	102	
stage										
	pTMN				0.374	T				0.194
	I	30	15	15		1	65	33	32	
	II	111	55	56		2	136	67	69	
	III	27	14	13		3	26	14	12	
	IV	2	2	0						
Lymph node metastasis					0.294					
	positive	63	35	28			N/A	N/A	N/A	
	negative	110	52	58			N/A	N/A	N/A	
Histology					0.521					0.594
	Invasive ductal	150	74	76			280	139	141	
	Others	23	13	10			33	18	15	
Histological Grade					0.433					0.07
	1,2	15	7	8			42	28	14	
	3	99	48	51			267	127	140	

	not reported	58	31	27		4	2	2
Race					0.669			
	White	102	53	49		N/A	N/A	N/A
	BLACK OR AFRICAN AMERICAN	55	27	28		N/A	N/A	N/A
	ASIAN	8	4	4		N/A	N/A	N/A
	not reported	8	3	5		N/A	N/A	N/A

Supplementary Table S3. Significant gene sets that associated to high expression of ANXA1 using Gene Set Enrichment Analysis (GSEA)(TCGA)

Gene Set Name	SIZE	ES[1]	NES[2]	NOM p-val[3]	FDR q-val[4]	FWER p-val[5]
HALLMARK_TGF_BETA_SIGNALING	51	0.6593	2.1881	0.0000	0.0031	0.0030
HALLMARK_TNFA_SIGNALING_VIA_NFKB	193	0.6371	2.1813	0.0000	0.0015	0.0030
HALLMARK_EPITHELIAL_MESENCHYMAL_TRANSITION	193	0.6933	2.1555	0.0000	0.0014	0.0030
HALLMARK_HYPOXIA	195	0.5159	2.1329	0.0000	0.0010	0.0030
HALLMARK_KRAS_SIGNALING_UP	190	0.5024	2.0277	0.0000	0.0047	0.0160
HALLMARK_ANGIOGENESIS	36	0.6510	1.9649	0.0000	0.0093	0.0350
HALLMARK_COAGULATION	134	0.5121	1.9642	0.0000	0.0080	0.0350
HALLMARK_UV_RESPONSE_DN	140	0.5317	1.9442	0.0000	0.0087	0.0430
HALLMARK_INFLAMMATORY_RESPONSE	194	0.5598	1.8843	0.0078	0.0146	0.0770
HALLMARK_APICAL_JUNCTION	194	0.4400	1.8539	0.0000	0.0192	0.1050
HALLMARK_APOPTOSIS	158	0.4248	1.8009	0.0000	0.0290	0.1540
HALLMARK_COMPLEMENT	192	0.4599	1.7553	0.0233	0.0391	0.2020
HALLMARK_IL2_STAT5_SIGNALING	193	0.4142	1.7211	0.0233	0.0467	0.2510

[1] ES, enrichment score; [2] NES, normalized enrichment score; [3] NOM-p-val, normalized p-value; [4] FDR q-val, false discovery rate q-value; [5] FWER p-val, family-wise error rate p-value

Supplementary Table S4. Significant gene sets that associated to high expression of ANXA1 using Gene Set Enrichment Analysis (GSEA)(METABRIC)

Gene Set Name	SIZE	ES[1]	NES[2]	NOM p-val[3]	FDR q-val[4]	FWER p-val[5]
HALLMARK_KRAS_SIGNALING_UP	189	0.6492	2.4215	0.0000	0.0000	0.0000
HALLMARK_TNFA_SIGNALING_VIA_NFKB	192	0.7142	2.3985	0.0000	0.0000	0.0000
HALLMARK_COAGULATION	128	0.6688	2.3712	0.0000	0.0000	0.0000
HALLMARK_EPITHELIAL_MESENCHYMAL_TRANSITION	191	0.7919	2.3509	0.0000	0.0000	0.0000
HALLMARK_APOPTOSIS	153	0.6113	2.3457	0.0000	0.0000	0.0000
HALLMARK_HYPOXIA	194	0.5964	2.2638	0.0000	0.0000	0.0000
HALLMARK_INFLAMMATORY_RESPONSE	190	0.6673	2.2433	0.0000	0.0000	0.0000
HALLMARK_COMPLEMENT	187	0.6308	2.2076	0.0000	0.0002	0.0010
HALLMARK_IL2_STAT5_SIGNALING	192	0.5580	2.1498	0.0000	0.0003	0.0020
HALLMARK_ANGIOGENESIS	33	0.7168	2.1103	0.0019	0.0004	0.0030
HALLMARK_IL6_JAK_STAT3_SIGNALING	82	0.6872	2.0758	0.0000	0.0006	0.0040
HALLMARK_TGF_BETA_SIGNALING	51	0.6101	2.0716	0.0000	0.0008	0.0050
HALLMARK_UV_RESPONSE_DN	142	0.5352	2.0201	0.0000	0.0013	0.0100
HALLMARK_APICAL_JUNCTION	188	0.4785	1.9148	0.0036	0.0061	0.0390
HALLMARK_ALLOGRAFT_REJECTION	183	0.6220	1.8311	0.0095	0.0130	0.0940
HALLMARK_P53_PATHWAY	188	0.4159	1.8221	0.0000	0.0132	0.1010
HALLMARK_PROTEIN_SECRETION	94	0.5077	1.7759	0.0174	0.0202	0.1350
HALLMARK_MYOGENESIS	192	0.4469	1.6937	0.0141	0.0401	0.2430
HALLMARK_INTERFERON_GAMMA_RESPONSE	186	0.6116	1.6848	0.0474	0.0413	0.2590

[1] ES, enrichment score [2] NES, normalized enrichment score; [3] NOM-p-val, normalized p-value; [4] FDR q-val, false discovery rate q-value; 5 FWER p-val, family-wise error rate p-value
